# The S-layer Protein DR_2577 Binds Deinoxanthin and under Desiccation Conditions Protects against UV-Radiation in *Deinococcus radiodurans*

**DOI:** 10.3389/fmicb.2016.00155

**Published:** 2016-02-16

**Authors:** Domenica Farci, Chavdar Slavov, Enzo Tramontano, Dario Piano

**Affiliations:** ^1^Laboratory of Plant Physiology and Photobiology, Department of Life and Environmental Sciences, University of CagliariCagliari, Italy; ^2^Department of Physical Chemistry, Institute of Physical and Theoretical ChemistryFrankfurt am Main, Germany; ^3^Laboratory of Molecular Virology, Department of Life and Environmental Sciences, University of CagliariCagliari, Italy; ^4^International Institute of Molecular and Cell BiologyWarsaw, Poland

**Keywords:** *Deinococcus radiodurans*, deinoxanthin, DR_2577, desiccation, quenching, UV light, radiation-resistance, S-layer

## Abstract

*Deinococcus radiodurans* has the puzzling ability to withstand over a broad range of extreme conditions including high doses of ultraviolet radiation and deep desiccation. This bacterium is surrounded by a surface layer (S-layer) built of a regular repetition of several proteins, assembled to form a paracrystalline structure. Here we report that the deletion of a main constituent of this S-layer, the gene DR_2577, causes a decrease in the UVC resistance, especially in desiccated cells. Moreover, we show that the DR_2577 protein binds the carotenoid deinoxanthin, a strong protective antioxidant specific of this bacterium. A further spectroscopical characterization of the deinoxanthin-DR_2577 complex revealed features which could suggest a protective role of DR_2577. We propose that, especially under desiccation, the S-layer shields the bacterium from incident ultraviolet light and could behave as a first lane of defense against UV radiation.

## Introduction

*Deinococcus radiodurans* is a pink-pigmented bacterium that appears in form of diplo- or tetra-cocci depending on the stage in the growth phase (Murray and Robinow, 1958; Battista, 1997). This bacterium is known not only for its extreme ability to resist from 20 to 200 times higher doses of ionizing radiation, UV radiation and deep desiccation if compared to *E. coli* (Anderson et al., 1956; Duggan et al., 1959; Mattimore and Battista, 1996; Battista, 1997; Battista et al., 1999; Cox and Battista, 2005; Fredrickson et al., 2008; Slade and Radman, 2011; Das and Misra, 2013) but also for being a model organism for surface layer (S-layer) studies (Baumeister et al., 1982, 1986).

The S-layers are paracrystalline two-dimensional arrays of proteins (Sleytr, 1978) which, irrespective of the cell wall organization (Silhavy et al., 2010), are equally spread among eubacteria and archaea (Sára and Sleytr, 2000) coating the external side of the cell wall (Sleytr et al., 1993; Bahl et al., 1997). Despite that the S-layer’s function seems to be variable, related to different ecologic determinants (Beveridge et al., 1997; Fagan and Fairweather, 2014) and frequently associated with cell protection (Sleytr et al., 1993; Beveridge et al., 1997; Rachel et al., 1997), the precise function for the *D. radiodurans* S-layer is not clear yet (Gentner and Mitchel, 1975; Karlin and Mrazek, 2001; Rothfuss et al., 2006). It was shown that S-layer enriched membranes from this bacterium possess a specific pattern of proteins (Farci et al., 2014) and a characteristic pink color due to the presence of deinoxanthin, the major carotenoid of *D. radiodurans* (Lemee et al., 1997). This S-layer is a complex assembly of several proteins organized in two regions: (i) a pore region where the main components are the protein DR_0774 (PilQ), a pilin assembled into dodecamers forming a type IV piliation system, and the protein DR_2508 also known as Hexagonally Packed Intermediate (HPI); (ii) an inter-pore region constituted by the protein DR_2577, also known as SlpA, a major S-layer component with a molecular mass of 124 kDa (Farci et al., 2015). Analogously to S-layers, functions of protection are typically reported also for deinoxanthin (Bing et al., 2007; Ji, 2010; Dartnell et al., 2012) and more in general for carotenoids (Naumov and Bokhan, 1984; Tuveson et al., 1988). However, in contrast to S-layers, in this case the main mechanisms associated to their functions are known in detail (Foote and Denny, 1968; Foote et al., 1970; Mathis and Kleo, 1973; Cogdell and Frank, 1987; Siefermann-Harms, 1987; Stahl and Sies, 2012). Carotenoids are a group of pigments with antioxidant properties which are equally spread between different groups of organisms (Moshell and Bjornson, 1977; Renger and Wolff, 1977; Krinsky, 1989a; Kobayashi et al., 1993; Moeller et al., 2005) with functions that span from the elaborate processes of protection from oxygen reactive species occurring in conditions of oxidative stress (Krinsky, 1989b) to the photoprotection related to photooxidation in photosynthetic organisms (Moore et al., 1982; Kirilovsky and Kerfeld, 2012; Leverenz et al., 2015). The *Deinococcus–Thermus* phylum, as well as several other groups of bacteria, shares the co-presence of S-layers and carotenoids (Tian and Hua, 2010). However, even if both may be important protection factors (Gentner and Mitchel, 1975; Karlin and Mrazek, 2001; Rothfuss et al., 2006; Dartnell et al., 2012), the functional data collected so far did not provide any evidence for a possible correlation between S-layers functions and the presence of specific carotenoids. In this study we report that *D. radiodurans* deletion mutants for the S-layer protein DR_2577 show a significant decrease of the UV resistance, especially under desiccation conditions. Furthermore, we show that the same protein binds the carotenoid deinoxanthin, a strong antioxidant with spectral features that could provide the base for the possible UV radiation-resistance mechanism. We propose that the S-layer, through the protein DR_2577, represents the first front of protection against UV irradiation especially in desiccated cells where the inner cell protection system is less effective.

## Materials and Methods

### Bacterial Strains and Growth Conditions

*Deinococcus radiodurans* strain R1 (ATCC 13939) was grown in Tryptone/Glucose/Yeast extract broth (TGY) (Murray, 1992) for 24 h at 30°C, with shaking at 250 rpm. For the UV resistance assay *D. radiodurans* R1 and ΔDR_2577 strains were grown in Tryptone/Glucose/Yeast extract (TGY) plates solidified with 1.5% (w/v) agar. The plates were incubated for 48 h at 30°C in the dark or in presence of a 9 W UVC lamp placed at a distance of 15 cm from the surface of the plate, reaching a irradiation rate of 2.75 J/s/m^2^ as described in Tanaka et al. (2005) and Dulermo et al. (2015). For the desiccation experiments, both cells strains (OD_650_ = 1.2) were processed as described in Mattimore and Battista (1996). Briefly, 1 ml volume of cells suspension was pelleted and resuspended in the same volume of 10 mM MgSO_4_. From this resuspension a volume of 50 μl was dropped on a glass slide and let desiccate in a desiccator under vacuum for 1 week. After this time the dried cells were exposed to a 9 W UVC lamp placed at a distance of 15 cm reaching a irradiation rate of 2.75 J/s/m^2^ (Tanaka et al., 2005; Dulermo et al., 2015) for 3 h at 30°C, while the controls for both strains were kept in dark. Finally both groups of dried cells, UV-exposed and not, were resuspended in the same initial volume of 10 mM MgSO_4_ and plated on a TGY agar. The DR_2577 deletion mutant, already described in Rothfuss et al. (2006), was kindly provided from Prof. Mary E. Lidstrom.

### DR_2577 Isolation and Deinoxanthin Extraction

The small scale procedure for the isolation and characterization of DR_2577 was performed as previously described (Farci et al., 2015). Briefly, cells were harvested by centrifugation of 1 liter cultures at 5000 × *g* for 10 min at 4°C and resuspended in 50 mM Na Phosphate pH 7.8. Whole cell membrane fractions were purified at 4°C as described (Farci et al., 2014). The membrane suspension was then subjected to a step of lysis with the French Pressure Cell, breaking the ultimate strong binding of the S-layer protein to the membrane layer, further digested with 100 μg/ml lysozyme for 8 h at 30°C under agitation (800 rpm), and then centrifuged (4°C, 48000 × *g* for 10 min) in order to obtain the protein DR_2577 in solution. The protein solution from four independent preparations was pooled reaching an initial volume of ∼30 ml and concentrated under flow of argon using an Amicon Stirred cell assembled with a 100 kDa cutoff membrane till a final volume of 200 μl. The protein sample was then loaded on a size exclusion chromatography column (Superose 6 10/300GL, GE Healthcare) previously equilibrated in 50 mM Na Phosphate pH 7.4, 0.06% (w/v) β-dodecylmaltoside (β-DDM). Pure DR_2577 samples obtained by this chromatography step were collected and precipitated by centrifugation (4°C, 4000 × *g* for 30 min) with PEG8000 10% in 50 mM Na Phosphate buffer pH 7.4. After centrifugation the supernatant was discharged and the pellet was dried for 6 h. Finally, the pigment deinoxanthin was extracted from the protein DR_2577 by pure solvents, polar (methanol, ethanol, and acetone) for the orange form or apolar (chloroform and hexane) for the pink form.

### Polyacrylamide Gel Electrophoresis (PAGE)

For denaturing Sodium Dodecyl Sulfate-Polyacrylamide Gel Electrophoresis (SDS-PAGE), 10% (w/v) separating polyacrylamide/urea gels with 4% (w/v) stacking gels were used (Schägger and Von Jagow, 1987). Samples were denatured with Rotiload (Roth) at room temperature before loading, and after the electrophoretic separation the gels were stained with Coomassie Brilliant Blue G250.

### Thin Layer Chromatography

Deinoxanthin purity was assessed on precast TLC foils (silica gel matrix on an aluminum support) with fluorescent indicator (254 nm). The mobile phase consisted of a mixture of 30% acetone and 70% chloroform. As a reference was used a total extract of thylakoid pigments from *Nicotiana tabacum* obtained by treating 100 μl of thylakoid membranes with 1 ml of 100% acetone (Haniewicz et al., 2013).

### Absorption Spectroscopy

The absorption spectra of the two deinoxanthin forms were recorded with concentrations adjusted to ∼0.8 AU in their UV maxima, while DR_2577 samples were measured at a protein concentration of 0.05 mg/ml protein. Measurements were performed on a Pharmacia Biotech Ultrospec 4000 spectrophotometer at 4°C in the range of 200-750 nm with an optical path length of 1 cm and a band-pass of 2 nm. Absorption spectra where recorded on an absorption Ultra Micro quartz cell with 10 mm light path (Hellma Analytics).

### Fluorescence Spectroscopy

The emission and excitation spectra of the deinoxanthin forms and DR_2577, concentrated as reported in the absorption spectroscopy section, were recorded on a Jasco FP-8200 spectrofluorometer at 4°C. Emission spectra in the range of 200–700 nm were recorded using the main absorption bands as excitation wavelength. The excitation spectra in the 200–700 nm range was recorded on the main emission band (325 nm). Fluorescence spectra where recorded on a fluorescence Ultra Micro quartz cell with 3 mm light path (Hellma Analytics).

## Results

### Deletion Mutants for DR_2577 (ΔDR_2577) are UV Radiation Sensitive

**FIGURE 1:**
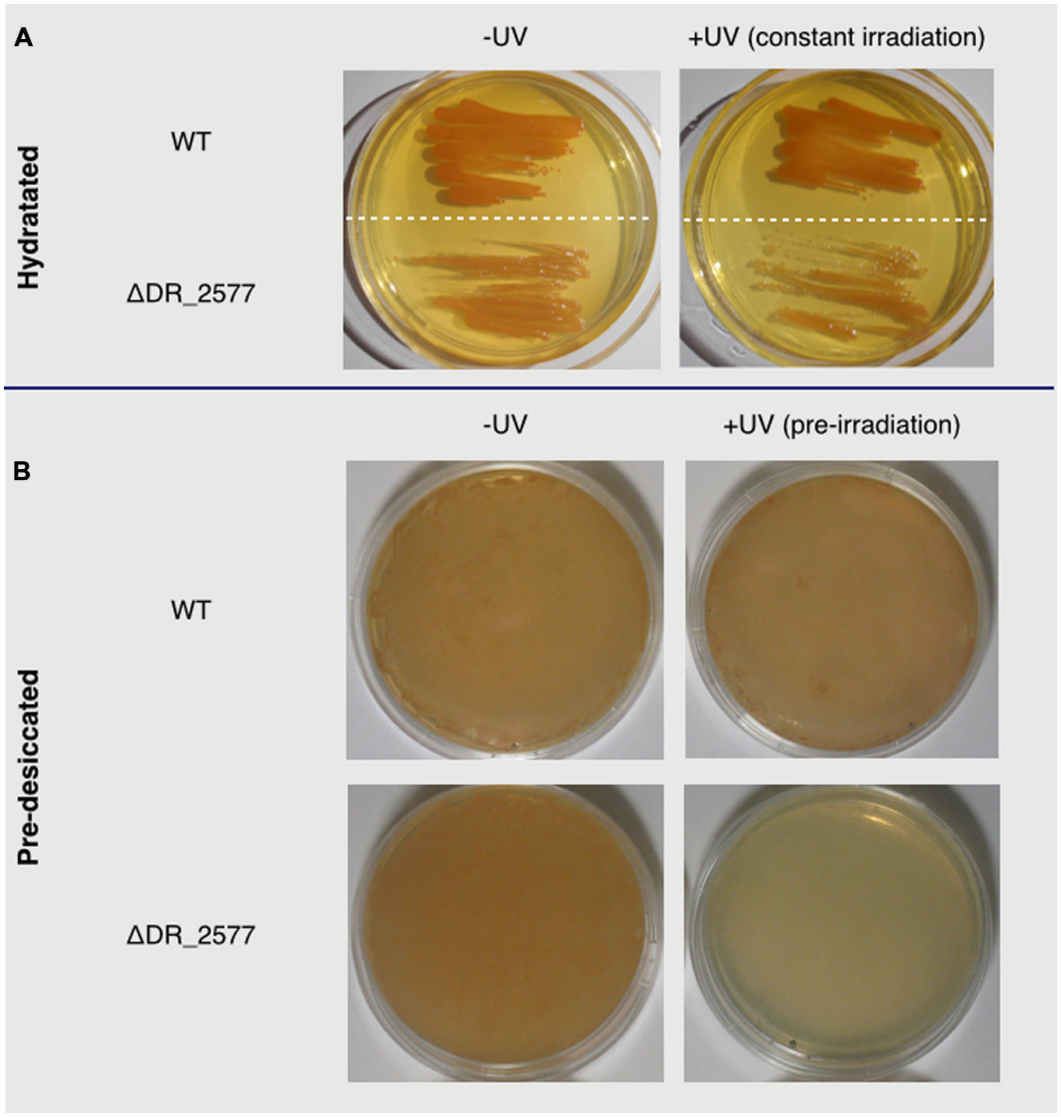
**In **(A)** are shown wild type and ΔDR_2577 strains grown under hydrated conditions in absence (left) or presence (right) of constant UV light; in **(B)** are shown the same strains grown after pre-desiccation (right) or pre-desiccation followed by a subsequent pre-exposure to UV light (left)**.

In *D. radiodurans* the S-layer protein DR_2577 is one of the main constituents of the cell wall (Farci et al., 2015) and is essential in maintaining the S-layer organization under conditions of chemical and mechanical stress (Rothfuss et al., 2006). Given these peculiar properties, we have asked whether DR_2577 is also involved in the characteristic UV resistance of *D. radiodurans*. With this aim we have cultured both the wild type and the deletion mutant for DR_2577 (ΔDR_2577) and compared their growth in absence and presence of UVC light (**Figure 1**). Under normal conditions the mutant growth was slightly slower with respect to the wild type, and this tendency was even more pronounced when the same tests were performed in presence of UVC (**Figure 1A**). Similar experiments, performed by pre-exposing the dried cells of both strains to UVC, showed a clear inhibition of the mutant growth (**Figure 1B**). These results suggest a protective role of DR_2577 against UV radiation particularly in conditions where the desiccation is a co-present factor of stress.

### DR_2577 is a Carotenoid Binding Protein

**FIGURE 2:**
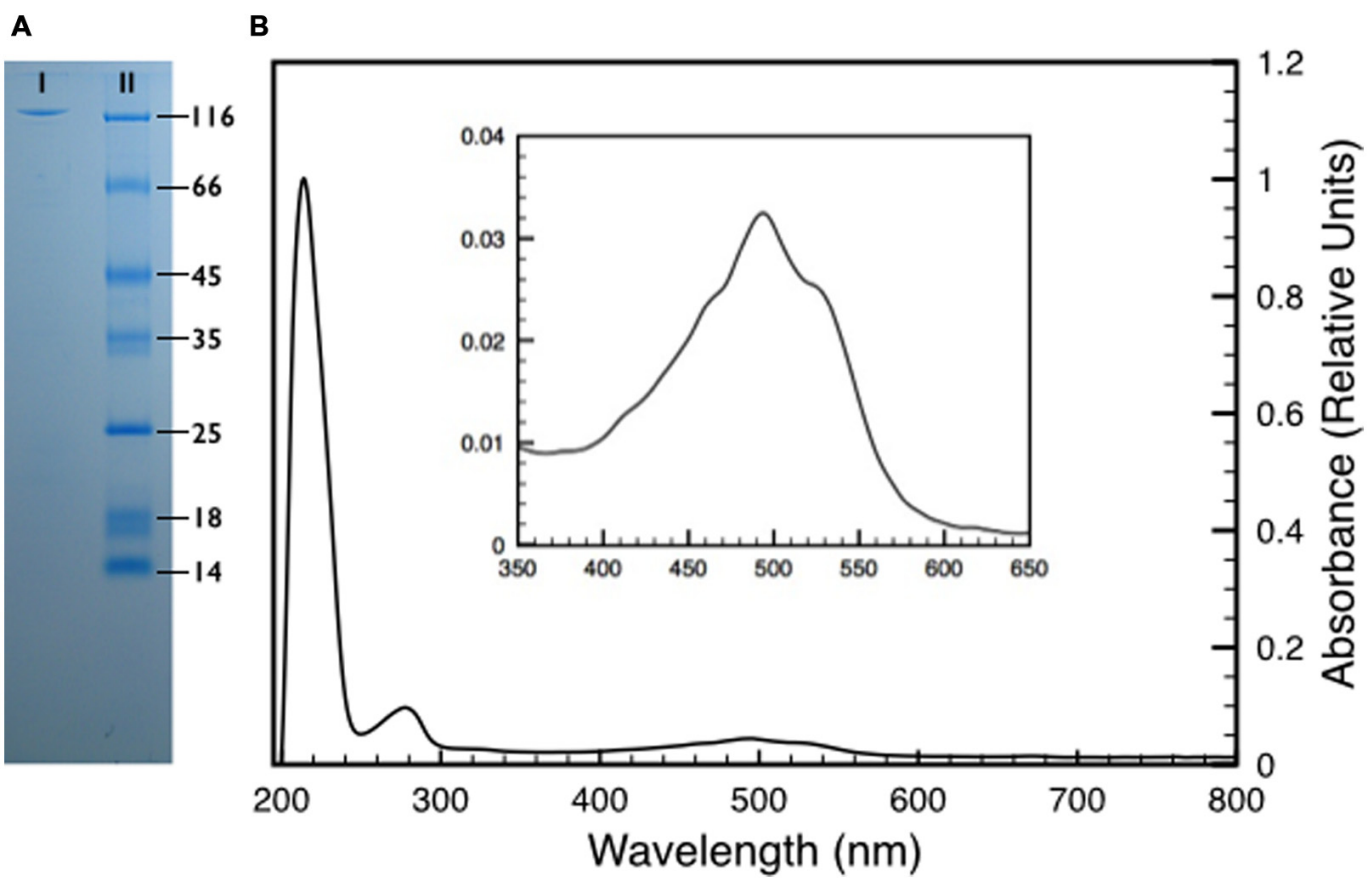
**(A)** Sodium Dodecyl SulphateSulfate-Polyacrylamide Gel Electrophoresis (SDS-PAGE) of a pure DR_2577 sample (I) compared to a molecular marker (II). The apparent weight of the DR_2577 band is consistent with its theoretical mass (123.7 kDa) when compared with the first band of the molecular marker (116 kDa). **(B)** Absorption spectrum of a pure DR_2577 sample in buffer. In the inset is shown a detail of the characteristic carotenoids-absorption bands in the region of the spectrum between 400 and 600 nm. See also Supplementary Figure S1.

To further characterize its contribution to the UV resistance, the protein DR_2577 was purified from isolated cell wall fragments as described (Farci et al., 2015). We improved this procedure, with the aim to scale up the protein production to a preparative grade (see experimental procedures), leading to pure pink samples of DR_2577. This result suggested a possible association between the protein and an unknown pigment (**Figure 2**; Supplementary Figure S1). In order to identify functional correlations between the pigmented substance and the S-layer, DR_2577 samples were analyzed by absorption spectroscopy (**Figure 2B**). From this analysis emerged a typical carotenoid signature in the visible region (400–600 nm) (**Figure 2B** inset) due to the S_0_→S_2_ transitions related to the polyene organization characteristic for carotenoids (Miller, 1934). Furthermore, an unusual and much intense absorption band in the UVC region (200–240 nm) was observed (**Figure 2B**). Generally, carotenoids have a less intense absorption in the UV with respect to the visible one (http://lipidbank.jp/cgi-bin/main.cgi?id=VCA) thus this characteristic appears to be a specific feature of the carotenoid bound by DR_2577. In order to clarify whether the anomalous UVC absorption band was due to the apoprotein or to the pigment, we have efficiently extracted the carotenoid from pure DR_2577 samples either using polar solvents (either protic or aprotic) or using apolar solvents. When the pigment was extracted by polar solvents (methanol, ethanol, or acetone) it turned from a typical pink color to an orange color, while extraction by apolar solvents (chloroform or hexane) was also efficient, but without a strong change in color since a clear pink solution was obtained (Supplementary Figure S1). As a further experiment, both extracts were resolved by Thin Layer Chromatography (TLC) showing that each of them is composed of a major species that, according to the references migrates as a xanthophyll, and a secondary component (Supplementary Figure S2). Taken together, these results suggest that the pigmented substance interacts with the apoprotein through non-covalent interactions, that it is a carotenoid, most likely a xanthophyll, and that it may occur in two different forms.

### Deinoxanthin is the UV-Absorbing Carotenoid Bound to the Protein DR_2577

**FIGURE 3:**
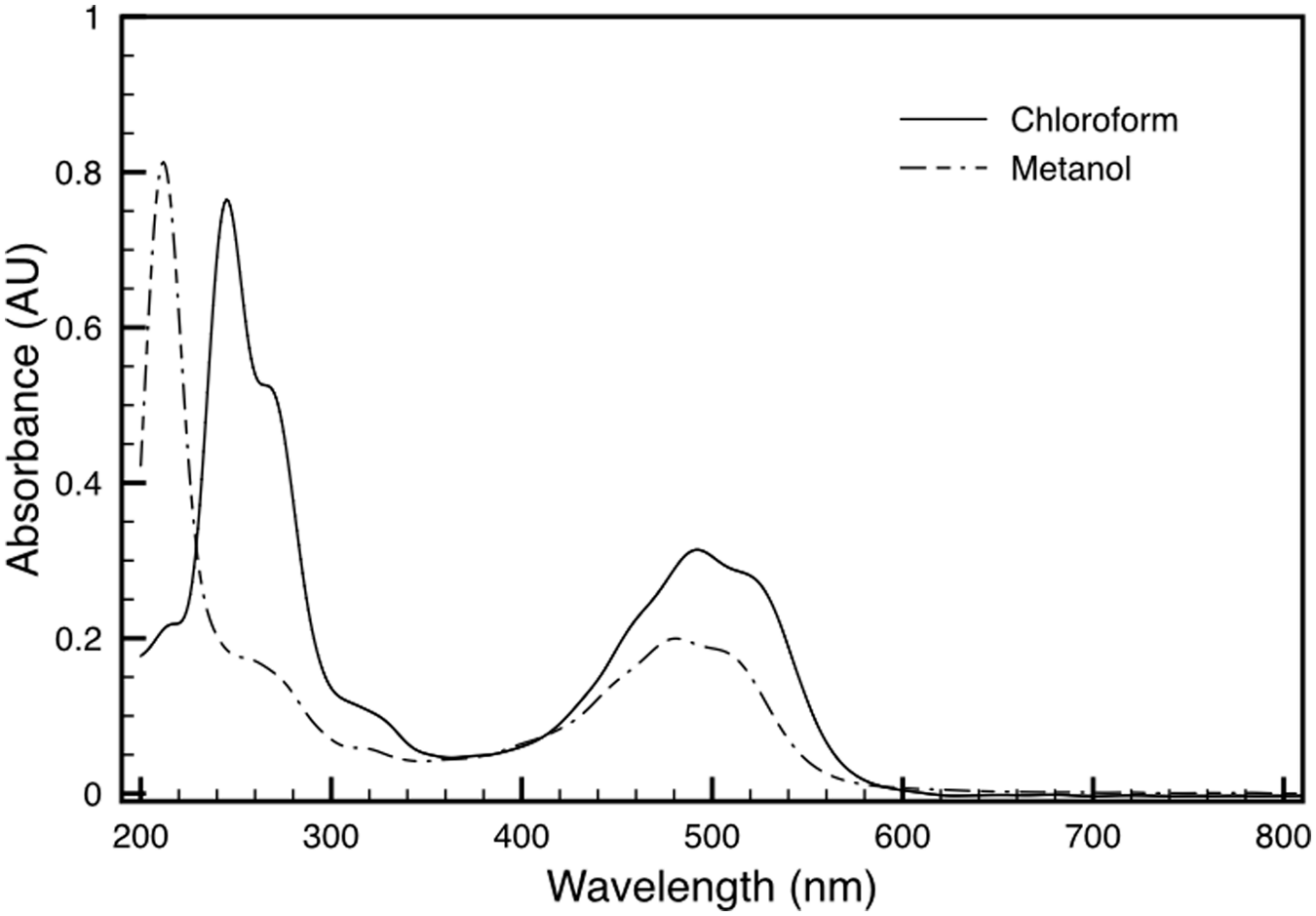
**Absorption spectra of deinoxanthin extract in chloroform (solid line) and in methanol (dashed line).** See also Supplementary Figure S1.

**FIGURE 4:**
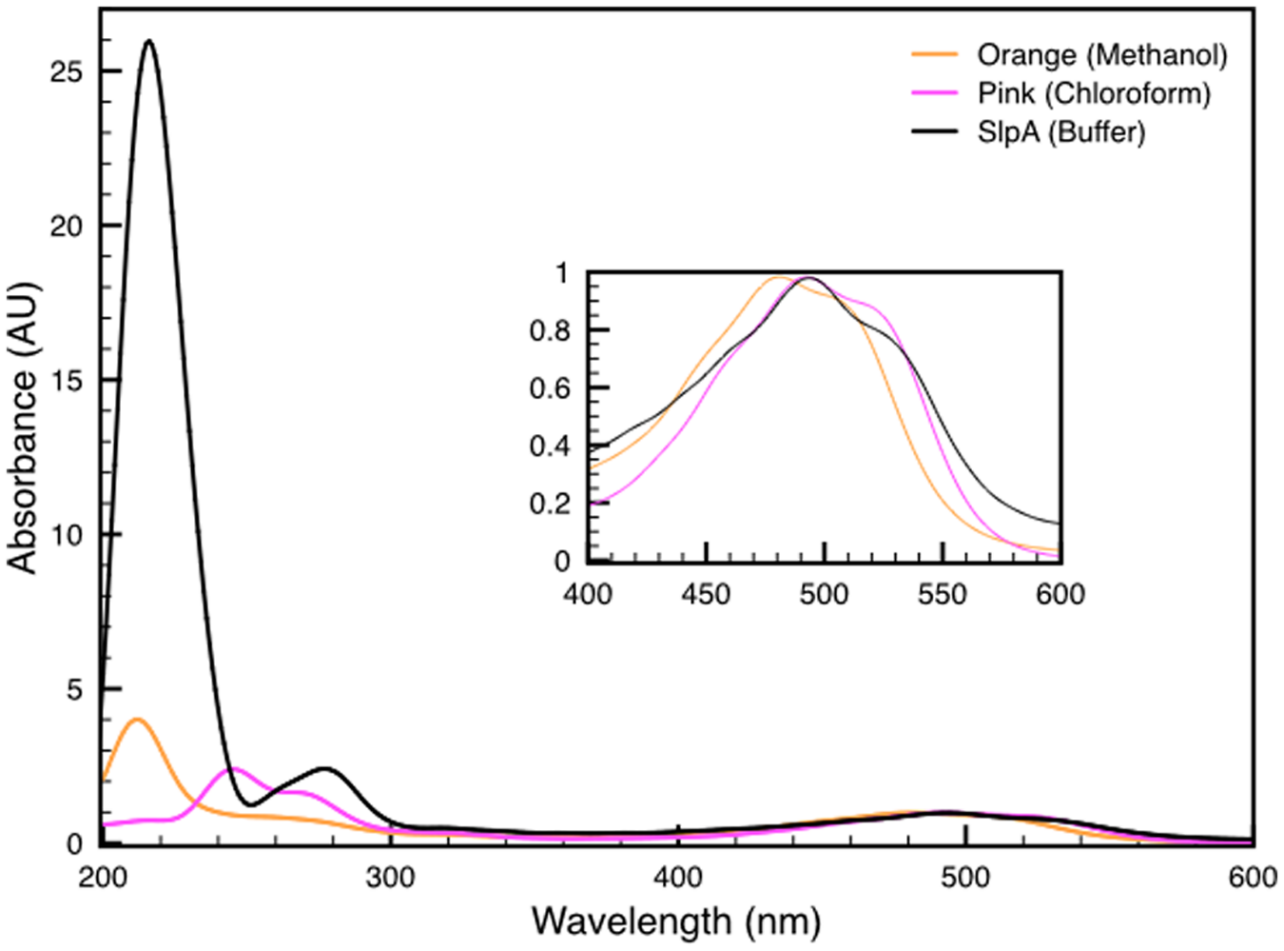
**Comparison between the absorption spectra of both deinoxanthin extracts in methanol (orange line) and in chloroform (pink line) with the spectrum of DR_2577 in buffer (black line).** In the inset is shown a detail of the characteristic carotenoids-absorption bands in the region of the spectrum between 400 and 600 nm. See also **Supplementary Figure S3**.

Both variants of the extracted carotenoid were investigated by absorption spectroscopy. When compared to the DR_2577 spectrum, the two spectra obtained kept the absorption in the UV range, which excludes a selective contribution due to the protein backbone (**Figure 3**). Considering the visible region, the spectrum of the orange form is shifted to shorter wavelengths as compared to either the pink form or DR_2577 (**Figure 4** inset). In a direct comparison of the three samples, the UV peak appears shifted to shorter wavelengths for both the orange form and the DR_2577 with respect to the pink form of free pigment (**Figure 4**). Moreover, the contribution in absorption of the UV peak was 4-fold and 2.5-fold with respect to the main peak in the visible region for the extracted orange and pink forms, respectively. In the case of DR_2577, the absorption in the UV range appeared to be amplified by several folds with respect to both extracted forms (**Figure 4**). Next, the absorption spectrum of the methanolic extract was compared to the spectrum reported recently for the pure deinoxanthin in the same solvent (Li et al., 2015); from this comparison it emerged that both spectra share a common pattern of bands in the carotenoids signature region (**Supplementary Figure S3**) confirming that deinoxanthin is the carotenoid associated to DR_2577.

### Emission and Excitation Properties of the Deinoxanthin-DR_2577 Complex

**FIGURE 5:**
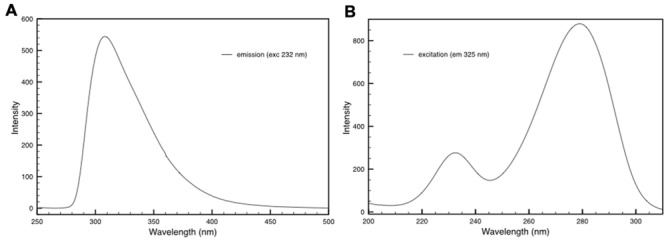
**(A)** Emission spectrum of DR_2577 in buffer with excitation at 232 nm. **(B)** Excitation spectrum of DR_2577 in buffer with emission at 325 nm.

The fluorescence properties of DR_2577 were also characterized. The DR_2577 samples were analyzed for their fluorescence emission and excitation showing typical features. First, the samples were investigated for their emission properties in the UV-Vis region presenting a single emission band with its maximum at 310–325 nm depending on the excitation wavelength (**Figure 5A**). Next, the same samples were characterized for their excitation properties, in order to associate the observed fluorescence with a particular UVC absorption. From this test emerged the presence of two spread bands covering a big part of the UVC region and having their maxima at 232 and 278 nm, respectively (**Figure 5B**). These measurements indicate that DR_2577 absorbs UVC light in a broad range of wavelengths (between 220 and 300 nm) emitting it in form of lower energetic photons in the UVA-Vis region (between 300 and 420 nm). The emission properties of DR_2577 appear to be well correlated with the emission properties of tyrosine. Being tyrosine highly represented in the sequence of DR_2577, it may take part in the emission properties of the protein and, together with deinoxanthin, in the possible quenching mechanism.

## Discussion

Up to the present work, neither specific localization nor association with a specific protein was reported for deinoxanthin (Lemee et al., 1997; Ji, 2010; Li et al., 2015), and so far the *D. radiodurans* S-layer had an undefined function (Gentner and Mitchel, 1975; Karlin and Mrazek, 2001; Rothfuss et al., 2006). In this work we show that the *D. radiodurans* S-layer plays an essential role in providing UV radiation resistance through the DR_2577 protein. Moreover, we show that DR_2577 binds through non-covalent interactions a strong antioxidant such as the carotenoid deinoxanthin. These findings could explain experimental observations previously reported in the literature such as the lack of radiation resistance in strains where the biosynthesis of deinoxanthin is knocked-out (Zhang et al., 2007), and the hypothesis that the high expression rate of the DR_2577 may have implication for the resistance to radiation damage (Karlin and Mrazek, 2001). Even if both are involved in the resistance against radiation or desiccation, so far a direct relationship between deinoxanthin and DR_2577 was not observed. The preliminary characterization of the deinoxanthin-DR_2577 complex, as reported for its two components taken separately, showed to have properties of radiation and desiccation resistance for which the fine mechanism of action still remain obscure.

Since the S-layer represents the first front of the bacterial cell that encounters damaging radiation, it needs to be capable of withstanding radiation damages during UV exposure. Accordingly, one of the main constituents of this layer, the DR_2577 apoprotein, is specifically designed to fulfill this function. The presence of UV sensitive amino acid residues in DR_2577 is strongly reduced: it contains only 2 tryptophanes, 5 histidines, and 2 cysteines (**Supplementary Table S1**). Nevertheless, about 10% of the DR_2577 amino acid residues are capable of strong UV absorption, i.e., 60 tyrosines (5.1%) and 52 phenylalanines (4.4%). The presence of phenylalanine and tyrosine residues in DR_2577, however, is purposeful since those amino acids are extensively reported as essential for the binding of carotenoids to proteins. In particular, the presence of phenylalanine residues along the polyene tail contributes significantly in coating the carotenoid cavities, while tyrosine residues are essential in the stabilization of the polar head (García-Martín et al., 2008). Such interpretation is also confirmed by the different frequencies of these four amino acids in DR_2577 with respect to other proteins (**Supplementary Table S1**).

Photooxidation in proteins occurs via two main pathways (Davies and Truscott, 2001; Pattison et al., 2012), a direct one and an indirect one. The direct damage caused by UVA and UVB radiation is typically associated with formation of triplet states and photoionization of the aromatic amino acids. The indirect pathway is linked to the formation of singlet oxygen and other reactive oxygen species. The photochemistry of aromatic amino acid residues (e.g., Tyr, Phe, Trp) is well described in the literature (Bent and Hayon, 1975a,b,c). One part of the UV light absorbed from those amino acids is typically reemitted as lower energy quanta (Tyr has a ∼14% fluorescence quantum yield -QY-, while Phe only about 4%), and another part is thermally dissipated to the environment. However, the major part of the absorbed UV light leads to triplet formation (QY of 40–50% for Tyr and Trp). UV damage of proteins occurs via the latter pathway (Bent and Hayon, 1975a,b,c). The triplet state of a photoexcited aromatic amino acid is typically quenched via photoionization, where an electron is transferred either to an oxygen molecule to form a super oxide radical ($O_{2}^{•–}$) or to disulfides (RSSR) to form a RSSR anion radical. Alternatively, energy transfer from the triplet-excited amino acid to oxygen could also occur, which leads to formation of singlet oxygen. All those pathways result in generation of dangerous reactive oxygen species and other radicals, which ultimately destroy proteins.

DR_2577, one of the main proteins of *D. radiodurans* S-layer, appears to be secured against these photo-oxidative damage pathways by the binding of deinoxanthin. This carotenoid has been investigated for its abilities to scavenge efficiently reactive oxygen species (Tian et al., 2007, 2009; Ji, 2010). Currently, the exact photoprotection mechanism of deinoxanthin in the S-layer is not known. However, similarly to other carotenoids (Foote and Denny, 1968; Foote et al., 1970; Krinsky, 1989a,b) and due to its extended conjugated π-system and the presence of hydroxyl groups, deinoxanthin can effectively quench singlet oxygen (Ji, 2010) and other reactive oxygen species (e.g., $O_{2}^{•–}$, RO^∙^, etc.) (Tian et al., 2007, 2009; Ji, 2010). During the purification of deinoxanthin, we observed that it can be extracted as a free pigment in two forms, orange and pink, depending on the solvent used for the extraction. Such a color change can also be observed depending on the redox potential and the pH of the solution used for cell lysis and DR_2577 purification. The presence of two redox states is also an indicator for the ROS quenching capabilities of the carotenoid. An important question, that arises based on this observation and should be addressed in future studies, is whether there is an active system that recycles or regulates the redox state of deinoxanthin.

Apart from mechanisms involving redox reactions, the protection role of deinoxanthin in complex with DR_2577, may be due to its strong absorption in the UV spectral range, which is unusual for carotenoids. These pigments are well known to quickly dissipate excitation energy as heat (Albani, 2004) and thus deinoxanthin contributes to direct filtering of impinging UV radiation. In addition, the close proximity of deinoxanthin to the strongly UV absorbing aromatic amino acid residues within the protein allows direct interaction and potentially excitation energy transfer. Consequently, this carotenoid in *D. radiodurans* S-layer may well be involved in a photoprotection mechanism similar to the one operating in the photosynthetic complexes (Olaizola et al., 1994; Ng and Pakrasi, 2001; Nath et al., 2013; Halac et al., 2014) with the peculiarity to be active against the UV radiation instead of the visible one. This model fits very well with a cell surface photo-protective mechanism where the quencher deinoxanthin is “caged” into a main S-layer protein scaffold. The properties imposed by the intrinsic organization of this structure would accomplish several requirements essential for the correct photonic of the system. First, the rigid orientation imposed by the protein scaffold (and the S-layer itself) would provide not only a very efficient light harvesting system, but also a subsequent system for an efficient quenching. Second, the apoprotein is not only making a scaffold for its quencher, but also contributes in complementing the light harvesting of deinoxanthin through its amino acid residues. Third, according to the position of this carotenoid-protein complex with respect to the whole cell, the chemo-protective mechanism based on the ROS scavenging would bring a significant decrement of efficiency due to the stericity imposed by the quencher rigidity if compared to the cytosol where the deinoxanthin have no need of specific constrains. The protection from ROS is extremely inefficient in case of desiccation (Mattimore and Battista, 1996), and the environments in which *D. radiodurans* is found are periodically characterized by dryness. Accordingly, considering that desiccated cells of ΔDR_2577 are significantly more sensitive to UV radiation, the deinoxanthin-DR_2577 complex would play a fundamental role acting as a shield for the incoming energy and decreasing the radiolysis damages, especially in dryness condition where not water molecules, but directly proteins are affected (Potts, 1994; França et al., 2007; Tian et al., 2007).

It remains to be clarified which of the above-mentioned protection mechanisms operate in the S-layer. However, what is clear is that DR_2577, as a major part of the regular paracrystalline organization of the *D. radiodurans* S-layer (Rothfuss et al., 2006; Farci et al., 2014), represents the functional unit of a highly specialized S-layer (Farci et al., 2015) that behaves as a shield against dangerous electromagnetic radiation. In effect, the S-layer of *D. radiodurans* is the protection forefront against the UV light. Certainly, the localization and the protection function of deinoxanthin is not limited to its association to the S-layer and DR_2577. In this and other works (Bing et al., 2007; Li et al., 2015) large amounts of deinoxanthin were also observed in the soluble fraction of the crude extracts and, at the moment, it cannot be stated whether in this cell compartment the pigment is present in a free form or it is associated to other proteins. However, association with DR_2577 can be excluded since this protein is isolated in the unsoluble fraction and kept in solution by non-ionic detergents (Farci et al., 2015). This is also confirmed by SDS-PAGEs analysis performed on the soluble fraction which did not indicate presence of DR_2577 in the cytosol.

These findings lead to important ecological and evolutionary implications. In *D. radiodurans* the presence of a UV shield associated to the S-layer is essential for surviving in extreme environments in which the cells would be exposed to dryness coupled with high and potentially dangerous UV doses. On our planet there are several ecological niches in which these conditions could occur. Environments such as deserts located at high altitude (Peng et al., 2009; Bouraoui et al., 2012) and the higher layers of the atmosphere (Yang et al., 2009) are examples of environments where Deinococcus species have been isolated and in which are observed high rates of UV exposure and periodical dryness. However, the development of such a well-specialized function may have originated in the early stages of life evolution, when our planet was characterized by a broad range of extreme environments surrounded by an anoxygenic atmosphere. This atmosphere could not filter out the incident UV light, exposing living organisms to strong and potentially mutagenic doses of radiation energy. In these environments S-layers as the one described in this work would represent an essential front for protection against UV irradiation, especially in desiccated cells where the inner protection system is less susceptible to react (García, 2011). This observation is also consistent with reports in which is shown a clear correlation between UV radiation-resistance and dryness (Mattimore and Battista, 1996) and in general between S-layer proteins and environmental protection during desiccation (Karlin and Mrazek, 2001; Kriško et al., 2010). Moreover, the antioxidant defense systems are inactive during water stress (França et al., 2007) and the increase in ROS formation drastically affects not only cell membranes, but also the general metabolism (Hansen et al., 2006). Accordingly, in certain extremophile bacteria and archaea the presence of highly specialized structures as some S-layers could be essential for their survival and development.

## Author Contributions

DP conceived the study, participated in its design and coordination, carried out the membranes and protein preparation, carried out the spectroscopical studies, participated in the biochemical studies and drafted the manuscript. DF participated in the design of the study, carried out the biochemical studies, participated in the spectroscopical studies, participated in the membranes and protein preparation and drafted the manuscript. CS participated in the design of the study, participated in the spectroscopical studies and drafted the manuscript. ET helped to draft the manuscript. All authors read and approved the final manuscript.

## Conflict of Interest Statement

The authors declare that the research was conducted in the absence of any commercial or financial relationships that could be construed as a potential conflict of interest.

## Supplementary Material

FIGURE S1 **Absorption Spectrum of Deinoxanthin.** Absorption spectrum of deinoxanthin extracted with methanol from pure samples of DR_2577 (black line) compared with the absorption spectrum of pure deinoxanthin (gray and red line) as reported by Li et al. (2015). In red are indicated the signature regions of the spectrum.

TABLE S1 **Amino acids frequencies.** The table shows residues occurring with anomalous frequencies in DR_2577 with respect to the averaged frequencies in proteins (King and Jukes, 1969). For comparison are also reported the amino acid frequencies of the other two main S-layer proteins, DR_2508 (HPI) and DR_0774 (PilQ), and of the Orange Carotenoid-Binding Protein (OCP_SYNY3) from *Synechocystis* (strain PCC 6803).
