# Supplementary figures and images for: The S-layer Protein DR_2577 Binds Deinoxanthin and under Desiccation Conditions Protects against UV-Radiation in Deinococcus radiodurans
Source: Front Microbiol. 2016 Feb 16;7:155. doi: 10.3389/fmicb.2016.00155 (PMC4754619; doi:10.3389/fmicb.2016.00155)

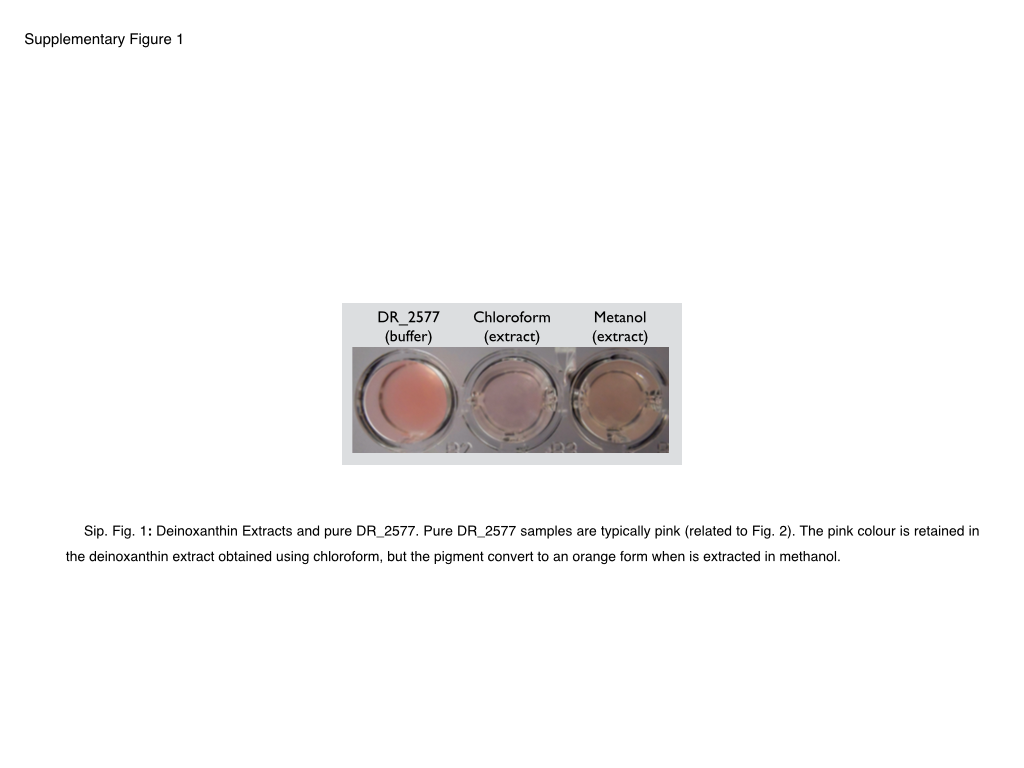

Supplement: Supplementary file 1 [file Image_1.TIFF]

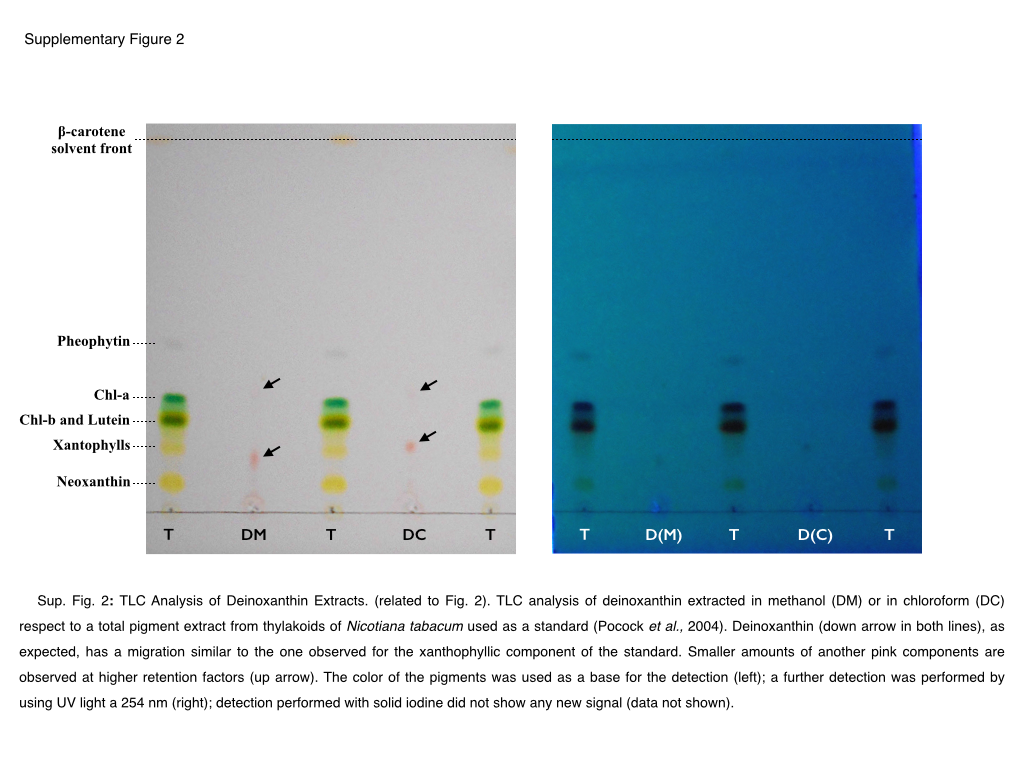

Supplement: Supplementary file 2 [file Image_2.TIFF]

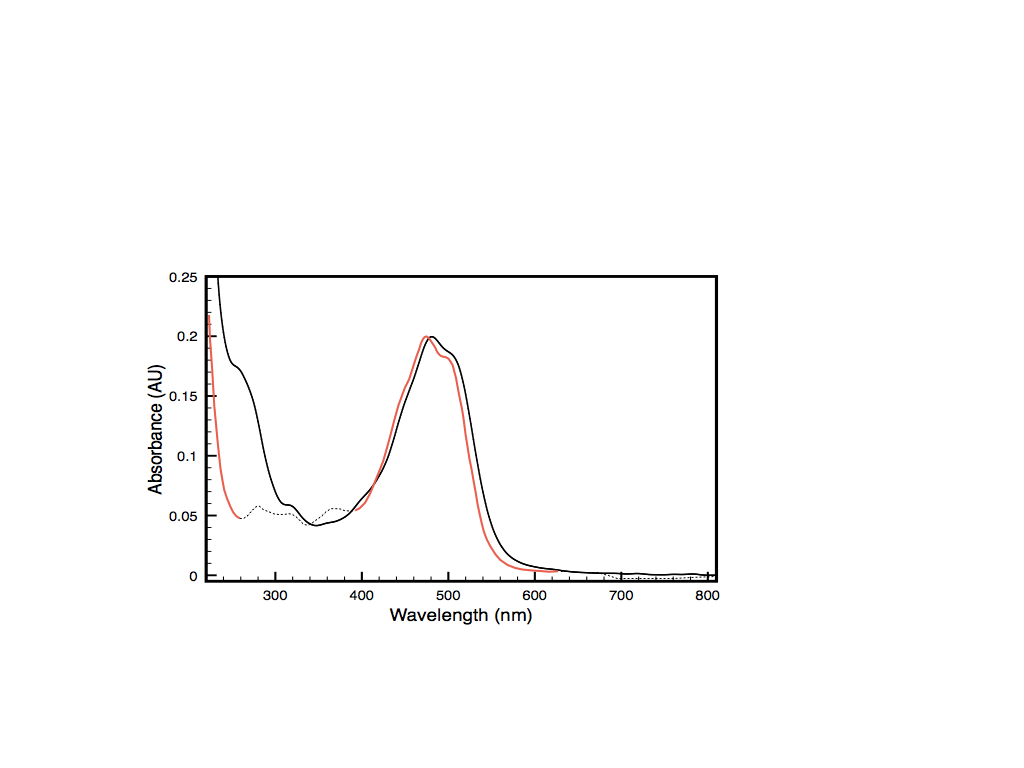

Supplement: FIGURE S1 — Absorption Spectrum of Deinoxanthin. Absorption spectrum of deinoxanthin extracted with methanol from pure samples of DR_2577 (black line) compared with the absorption spectrum of pure deinoxanthin (gray and red line) as reported by Li et al. (2015). In red are indicated the signature regions of the spectrum. [file Image_3.TIFF]
