# Supplementary material for: The S-layer Protein DR_2577 Binds Deinoxanthin and under Desiccation Conditions Protects against UV-Radiation in Deinococcus radiodurans
Source: Front Microbiol. 2016 Feb 16;7:155. doi: 10.3389/fmicb.2016.00155 (PMC4754619; doi:10.3389/fmicb.2016.00155)
Supplement: TABLE S1 — Amino acids frequencies. The table shows residues occurring with anomalous frequencies in DR_2577 with respect to the averaged frequencies in proteins (King and Jukes, 1969). For comparison are also reported the amino acid frequencies of the other two main S-layer proteins, DR_2508 (HPI) and DR_0774 (PilQ), and of the Orange Carotenoid-Binding Protein (OCP_SYNY3) from Synechocystis (strain PCC 6803). [file Table_1.PDF]

	S-layer proteins (*Deinococcus radiodurans*)*				orange carotenoid-binding protein (OCP) (*Synechocystis* strain PCC 6803)*
**Amino acid**	**Average (%)**	**DR_2577 (%)**	**DR_2508 (%)**	**DR_0774 (%)**	**slr_1963 (%)**
Phenylalanine	4,00	4,40	3.7	3.8	5.7
photo-damageable amino acids: Tyrosine	3,25	5,10	2.7	1.8	1.6
photo-damageable amino acids: Histidine	2,90	0,43	0.1	0.3	0
photo-damageable amino acids: Cysteine	3,30	0,17	0.7	0	0.9
photo-damageable amino acids: Tryptophan	1,30	0,08	0.7	0.3	1.6

***frequencies are calculated using the software ProtParam from the Expasy platform (http://web.expasy.org/protparam/).**

- (green) = UV absorption and carotenoid binding residues
- (ochre) = UV absorption, carotenoid binding and photo-sensitive residues (UV)
- (red) = photo-sensitive residues (UV-Vis)
- (purple) = photo-sensitive residues (UV)
